# Supplementary material for: Off-target piRNA gene silencing in Drosophila melanogaster rescued by a transposable element insertion
Source: PLoS Genet. 2023 Feb 21;19(2):e1010598. doi: 10.1371/journal.pgen.1010598 (PMC9983838; doi:10.1371/journal.pgen.1010598)
Supplement: S1 Fig — (PDF) [file pgen.1010598.s001.pdf]

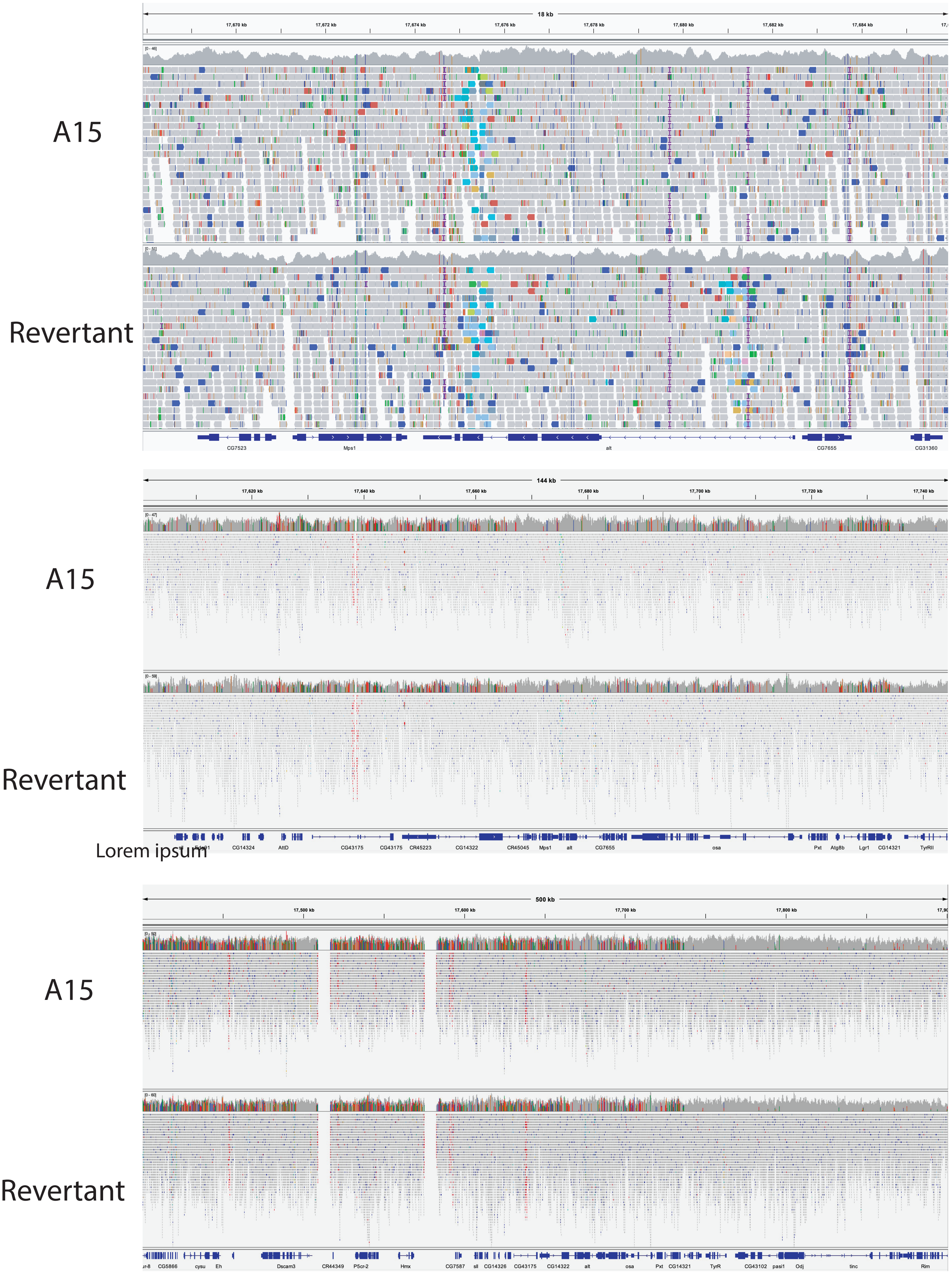

Supplemental 1. Coverage plot of reads for *Mps1, alt* and flanking region for A15 and Revertant Strains at various spans: 18 kb (top), 144 kb (middle), 500 kb (bottom).
