# Supplementary material for: Off-target piRNA gene silencing in Drosophila melanogaster rescued by a transposable element insertion
Source: PLoS Genet. 2023 Feb 21;19(2):e1010598. doi: 10.1371/journal.pgen.1010598 (PMC9983838; doi:10.1371/journal.pgen.1010598)
Supplement: S2 Fig — (PDF) [file pgen.1010598.s002.pdf]

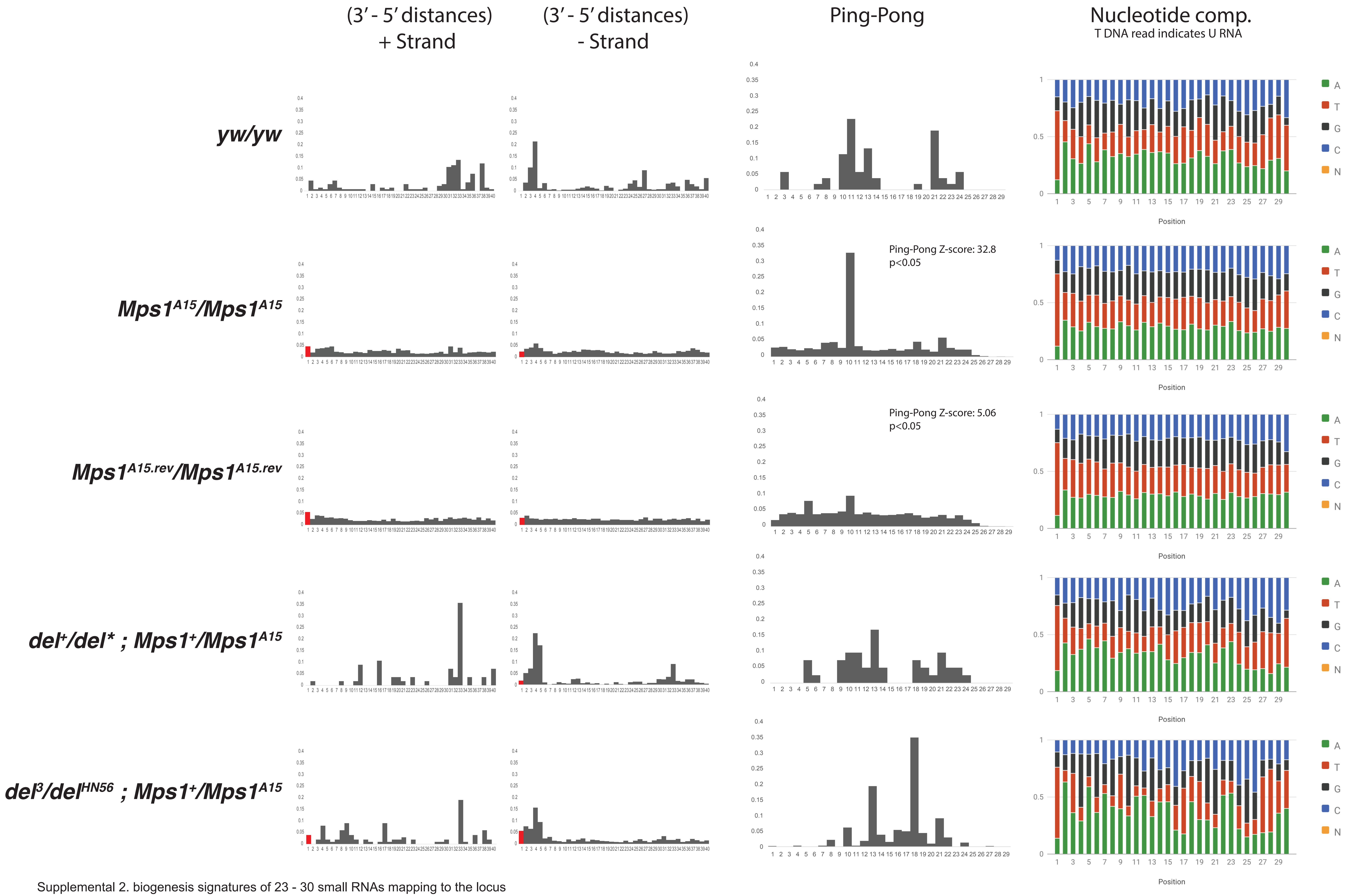

Supplemental 2. biogenesis signatures of 23 - 30 small RNAs mapping to the locus

Note: For two genotypes (del[+}/del[*]; Mps1[A15]/Mps1[A15] and del[3]/del[HN56]; Mps1[A15]/Mps[A15]) library size precluded reliable analysis of ping-pong and 3'-5' distance. For this reason, they are excluded..
