# Supplementary material for: Off-target piRNA gene silencing in Drosophila melanogaster rescued by a transposable element insertion
Source: PLoS Genet. 2023 Feb 21;19(2):e1010598. doi: 10.1371/journal.pgen.1010598 (PMC9983838; doi:10.1371/journal.pgen.1010598)
Supplement: S3 Fig — (PDF) [file pgen.1010598.s003.pdf]

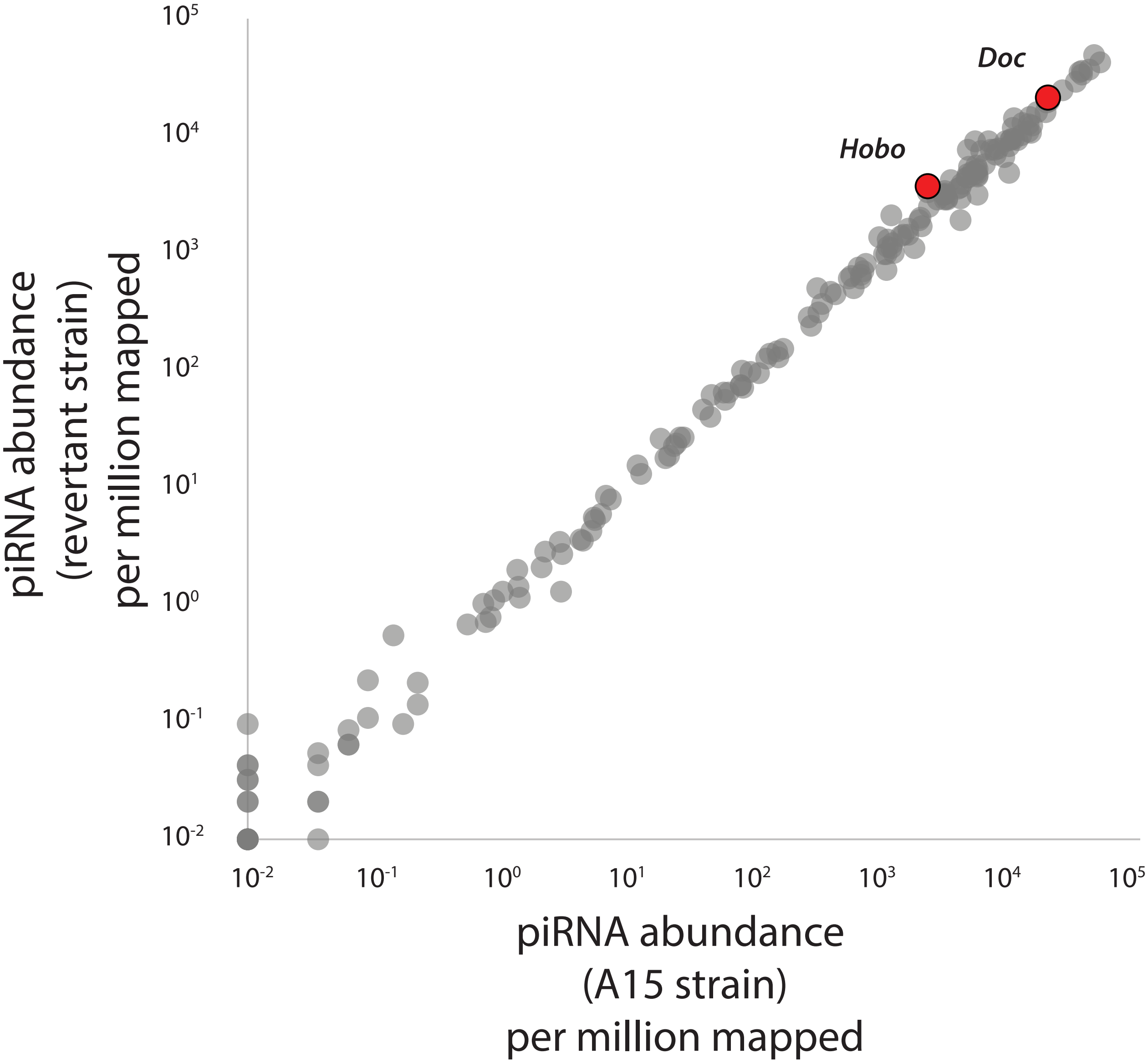

Supplemental Figure 3. piRNA coverage (per million mapped) of Drosophila transposable elements. Elements show very similar piRNAs abundances across strains. In both strains, piRNA abundance from the Doc element is about 10 fold that of piRNA abundancefrom the Hobo element.
