# Supplementary material for: Off-target piRNA gene silencing in Drosophila melanogaster rescued by a transposable element insertion
Source: PLoS Genet. 2023 Feb 21;19(2):e1010598. doi: 10.1371/journal.pgen.1010598 (PMC9983838; doi:10.1371/journal.pgen.1010598)
Supplement: S4 Fig — (PDF) [file pgen.1010598.s004.pdf]

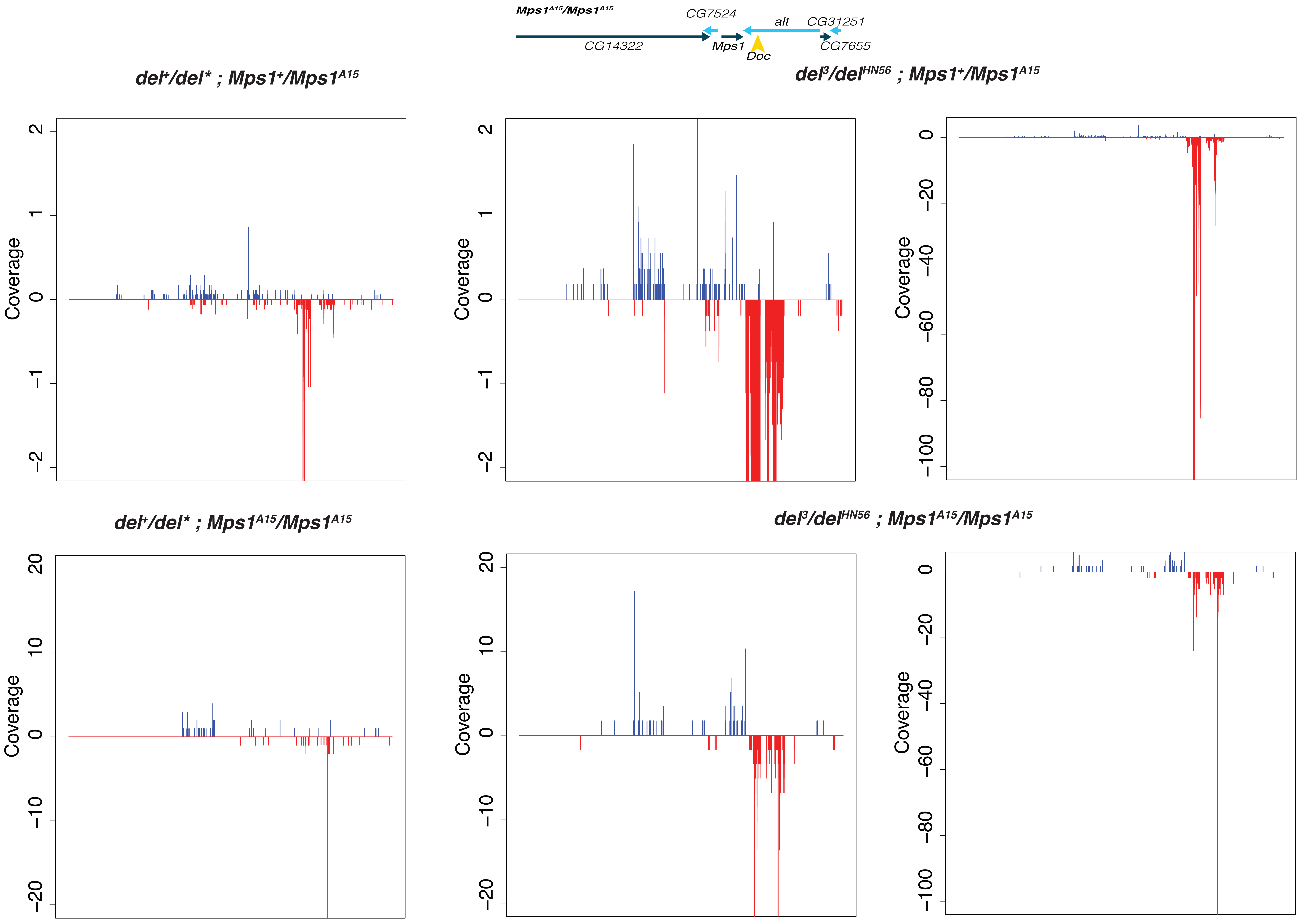

Supplemental Figure 4. piRNA coverage (per million mapped) of the span from CG14322 to CG31251 for all four combinations of deadlock and A15 genotypes (heterozygous/homozygous). Deadlock homozygous mutations lead to the production of a large excess of - strand piRNAs in the vicinity of the Doc insertion. Far right are shown rescaled mappings to reveal this on the - strand. A15 heterozygote genotypes (top row) are shown at a closer scale (-2 to 2) compared to A15 homozygous genotypes (bottom row) that have a greater abundance and are shown at agreater scale (-10 to 10).
