# Supplementary material for: Off-target piRNA gene silencing in Drosophila melanogaster rescued by a transposable element insertion
Source: PLoS Genet. 2023 Feb 21;19(2):e1010598. doi: 10.1371/journal.pgen.1010598 (PMC9983838; doi:10.1371/journal.pgen.1010598)
Supplement: S1 File — (DOCX) [file pgen.1010598.s005.docx]

Note:This is reverse complemented with respect to standard orientation.

TE Insertions are same orientation.

CAPS: *alt ge*ne Span

**CAPS.BOLD: *alt* TRANSCRIPT**

**CAPS.BOLD.UNDERLINE: *alt* CDS**

***DOC*, or *HOBO*: Insertion**

GCCGCTGA: TSD

AGCCTTTATGAGTCACTCCA: Primer A of Primer Pair 1

TAATGAATGAGTGCGAGTAC: Primer A of Primer Pair 2

**AGGCTAAGCTTCGCGAACTA:** Primer A of Primer Pair 3

R6.45 – 17,682,515 –17,673,732

**GTATGTCGCCACGGTCACTCTGTTTGCAAATGTTTCAATTTTCCCACTAACGAAAAAGTGTAAGTCCGCAGCCGAAAGGCATAAGAAAGTTGAGAAAAAGTTTAACAGTTGCAA**GTAAGAACTGGTTTGATAAAGCGTATCTAAATCCAAGCAAAGTGATGAAAAAACTGTCGACGCGACAACCCAACACACACACACACTCAAACAGGCGCAGCGCTCCGGCACACATACACAAGTGGCTTGCAAATGGCGGATGCGTTTCCAACAAAAAG**AAAAGAAGAAGGAAAAGAAAGACTGCATTGTAAATGTGAAATGTTGTGAAAATTGTTGACGTTGCAAGTGGCTTAAAATCGGCTGAAAATCAGCGTTAGAAGTATAGAGACTGGAGATAAGAAGGGGAGAGCGGTGTACATGTATGTGT**GTAAGTGCAGTGCGACGCCCTTTTGTCTTGAGAGGAAAAAGGAAGCCACTACTTTATTTATCGGTGTGCAATTATTCAAGGTGCCAATTAGTAGTCGACACACTTTCACTGCTCCCCATTGCTTAATTATGAGCAAAGGGGACAGCTGACTGCTTTGATTTGCCAAAAATCTCGGGCGCCGCCGTCTGGCCGGGGCCCTGCCCCCGCCCTTTCCCATTTGTTGTTCGCTGATTTTATCAAAGAAAAAGACAAGAAATGAGCATAATTGATGCGCTGACCTCACCACACACACACACATAAGCATATGCGGGCTTATCAAAGGAGCGAGAGCGGAGCAGAGCTCAAAAGAGAGAGCGGAGCAGAGCAGCTGCAGTCCAATAAATCATAGACTCAAAAATCGTTCGTCATCGGGGAAAACCAATTGACAATCAACAAATGCTATCGACGTCTACGTCAGCAATCAATAATCATTAACCGCCAGCTTTAGACAATATCAATTTGCCTATTTATAGCCTTTATGAGTCACTCCACATTTTAGACCAACCTTCCATCTGTGGACCAAAAGAAAAAATTAGGGAAACGAACTTGGAACCTGACGCTCCCACGCAGGAATAACAAGAAGACACCACACACCGCAAAAAACATGCCACGCTTTCTTTCCCACAGAGATAAATAAAGCAGCATTGCATGTTGCATAAATATTACGGGAGAAAAAGACGAAAAGTAGGGAGCAGTGCTTTCTAAAATTAAAACTCTTGA***HOBO***GCCGCTGAAAATAGCAGGGGCAAAAAATGCATTTATGAATAAATAGATGAAAAATAATGAATGAGTGCGAGTACGCTCTCGCTCAGCGGTGACAGAGAGAGCGAGGAAAGCACTTGCACCGTTATCAGTGATGCTGCTGCGAAAGCAAGCTAGTAAAAGTATAATTTCAAATTATTTTTTTATGAAAGAACAAAAATAATATAAAATGGAGAACCCGGTTAAATTTTAGATAGATTGGAAGTTTTCAGTTTTAAGAAGTTATTAGGATATGTAAGTTGATTATGAAAAACAATTTTTTTTTAGAAACAAATCTAGACATTTATAATTCGCATAAGGACTTTAATAATTATACACAGTTACGAGGATCAATAACTTTTTTATTTCCATTTGAAGAGATGTAATCGCATTGGCGAATACTTGACCCACTTCAAACACGCTGCCTTCAAAGGCCAGAAAATGGTCAACTTGGCATTTCTGGCCGGGGCTCACCAACAGGGGAGAAATTCCATGTGCCAGCAAGTGCGAGTGTGTGTGTGAGCAGGCAGCTAATTGAACACTGGGGCGCCGCAAAAGAAAGGGGGAGGGAGGGAAGCAACGGCACAGAAAGATAGAAAGAAAAACGGGAAAATTAGGCCAACTGTGCAGTTCGATGTCAGTTTGCCAGTTTGTCAGTTGGCTGTAACGAGGCCGAGCAGCCAGCGTGTTTTGCATGCTGTTCGACCATAATACATAATTATTTATAGATGGTCATAATGATAATTGGCAGCAAGATTTCGACTTTCACACACTCGTTTATACAAAAACTCACACAAAAACACTGGCCAGTCACACTCACGCACACATGGGCGCTTTTCTTCCGGTTGATGACCTTTACCGGCGTTTTTATTGTGCTTTTTATTCTCTTTTGGCTTTGGCTCTCTTCTGCCGCCACCGCTTCTGCTGCTTCTTCCTCCCCCGCCTCTATACTCACGGAATCGGAATCCGCAGCCCGGACTTTACTTACTTTCCGTTAAACGCCATCGATTTGTTTGACGGAAACCCGTTTCCATTGTGCCATTAAATATTAATTTAACTTTGTGGTCAGTCCACCCATCCGTAATAATGAATGTCGGTTTCATAGGACTAGATAATAGACAATTGGAGTTGTAAAAACTTTCATAAATTGTAGTGAAGATGCTATGAACCATCTAAATTGTCACAATGGTCGCAATACAACAGTTGGAAACACTAAAAATGGTTACATTTGTTAATAAATGAAATCAAGAGTTAGTTATATGTAGATAGAGTAAACCTGGAAAGATTTGCTGTATACGGATTATTCATCTACCCAGTATCACGTAACCCAGTATTTTTGAAAGCCCCTTAGAAATGTTTGTTGATTGTGGATAAGAAGAAACAGAAACACAGTCAGTATCTTTTAGCCAGAAACATGACGCGAGCCAGCAAAGCAGCAGAAATA**AGAAGCCAGAACTTGACAAGCCAACAGAGGAAAATAAATACCCGACCTGCCAGTTATCGCAGCCGCAGAGAGCAACAACCTTGAATTGAAATTTTTGCAATTTGTGAATTCTTCCACTGAGCTCGCGGAGTCAGCGAAATTTCAAGTTTTGTTCTAACTGTGGTGGGAGCGAAGAGAGAAAAGTGCCACGGAGCGCAAGCACACTGAGAATAAAACGTTCTGTTAGTAAT**GTTAGTATTTTTTATTTCAAAAATGTACAAACTACAAATATATATTATTTTAAAGTTTCAAAGATAACTGGGCGGAGTAAATATATAAACGTATTTTATTTTGATGATACAAGTAATGTTATCGCATATTTTAAACTCAAGACTTATTGCAAATTTGTTTGCTGATTCTTCAATAAAATCTTGCTCTCATAAAACGTAACTTGCACTTACAGAAAACATCCAGAAAATAACCTATTCAAAAATAAATATTTAGTCGATCTTGGGAATTGCTTCAAGTGTAGAGAGCTGAGGAGAGATCTCGAATTGCGAGCCCAGCTCTGCTGCGAATCTTTTCACTAGCCTTTGGCATTCGCATCGTG**TCCATTGGAAGAGACTCGTGGAGTTTAACGAAAATAGATTAAACAGTGTTGATTTTTTTTGCGAATTGGCAAATTTGCATACGTGTGCGTCAGAATCTAGACTTTGAGTTATTTTAAATTGTTATTCTTTTTTCGGATTGTTAT**GTGAGTTCCTTTCAATCTGCGTCAACACCTTTGGGTGCTGGAAATGTCTACCAACTTTCGTTCAAGGCTGCCCAGCAGCTGCTGTTTCATAAGCTAATTTTATTCTTTGTGACTTTGTCGGCTCCCCACTTCTGCCCGGAATACATTTTTCCGTCTTAATCGCTGAGTTCTTTGAGGAATCGATAATTCCAGCTGAGATTTCTTATCGCCCTTCCTGCGCCAAATGAAATTCAAATGACCTTTTTGCCTAAATTTGAAATTCATGGGGATTTATTGGCGAGTTGAGCTGTATAATCCGGAATACCAGGAATATTGAGCCATTCAGCAGTGCATTTTTTTTATCTCAGCAGAGCAACAACAACAATGAAATTATCAACCGCACAGCGTTTTTATTAATATAACGTCCAGCAAGCCCAGTCAACTGCAGCAATAACAACGTG**TTCATACAAAAGGAGAACTTGTGTTGTTGCGCTCTGAACCATTTTCCGGCCG**GTAAACCGATAAGCACGTTCAACTCAAACAATCCGGCTATATAAAGCGTAATTGCCATTGAATGTAGTGTCGTTGATCTGTCGTTTTTAGTGGGCATTTCTTCGGCGTTTTTCGATTTTTGACCCCCACGTCATCAGTTTATACTACATTGTAGTGGTTCTCAAGCTGAAAAAAGCTGATAATGCTACGAAATGAGTTTGATAGCAAAAATATATACACGAGTGACCCTAAAAAGCATTTCTAACATTTCCACACTTGAGATTATTTTTTGCTTTGTTGGTTGCTTTCCTTGGAAACCAAACGGAGAAAACGTGGCCGTAATTCCGGGCTTCCCCTTTTAGATGAAATCGAACATTATTGTTTAATTTTTTCATATGTATTTACACCTGCAAATGTAAATACCTAGAAATATTTACCTTCTAATATTGCTAAAATACAAAATAGTAACGAGAAACTCTCTATTTATTCAACAG**CATTCGCAAAGGAGATCCCTGAGAAAAATCCACGGCTAAACCGGAAAGATATGCAAATATGGACTTCCACATACTGATCGTCATCGGTTGTGTGGTCAGCGCCTCGCTGCTCTCGTTCCTGTTCATCAACAAGATCTTCCGGCGCAAGACTTTTGAGGAGGTGGTAGCCGAGAAGCGTGCCCTGAGCGCCAATCTCTACAAGGCGGCCGGTGGTGCCGCTACCAAGAAGCCCAAGAAGAAGGAACTTAAGCGCGAAAAGAAGCAACGTCAGCGGGAACAGCAGAGGGATGTGAACAACGAGCCGGAACCAGAGGAAGCCGAAGACTACTCCGATGGTCAGTCGGAGGGTCAGGGCTCCGTGGCTGGCGAGGAACCCGGTCTCTCCAAGCAGCATGTTGAATTTGAACCCGATGCAGAGGTCCTCACTGATCAGCGACGACCCAGTAGCGTGGCTGAGAAGGAGAACCAACCTTCTGGGGCTGGCAAAAAGGGAAAGAAGGATAAACGTAACGGAGCCAACAACAAGACAGCCGGAATCCTGGTCAACAAGAACGAAACTGTTGCTGTTAAGCCTGCAGCTAACGCCGAGGAGACACCAACTTTGAATAATTTTGAAACCAAGATCCCCAAGGATGTGGTGGAGCTGAAGAAGCAAGAGCAAAAGGAACGCAAAGAAGACAACAACAACAAGCAGCAAAGTCAGAAAAAGATCGCCGGAGGCAATGTGTCCAAGAAGGAGAAGGTCGTAGCTGTCGAAACAGAGGCACCCGTAGTGACCAAGCAGATCCTCAAGCAACAGCAGCAGCAGCAGAATGGCTCACCCAAAACGCAACACAACCAGGCCAACAACAAAACCAAGCAGCAGCAGCAAAACAAGAAACAGAATCAAAAGGAAACCCTAACGGGTAAGGATTTGGCTCATGCTTTGGATAAACTGGCCGATCACCAGAACCAGACCATTGGTGTCAACGCTCTGATGAACGTATTTTCTCGTGCCGAGCTAAATCGCTCAGAGATTCAGATACTCATCGACTATCTGCTAAACAAGCAACAGGATATGCCCGCTTCGCACTCTGAGTGGTCGGATGACATCTGCCAGAAACTCAAGCGTCAGCTGGAAGAGAAGGAAAAGCTGTTGGCCGAGGAGCAGGAGGCCTCCATCGGAATTCAGGCTAAGCTTCGCGAACTACGCCAAGAGGTTAACACAGAACGCGCCCAGATGCATGCCCGAAACCAGGCTTATATCGATAAGTTGCAAGGCAAGGAGCAGGAGCTAGCCGCCCTCAACCAGGAGCTGTCCAGTCTGAATGACAAGTTGACGCTTGAGCGTCAACAGCTTACG**GTAAGAAAAGCTTCTGAGATTTATAAGAATATTTTCAAACATAGCCTATATTCAAATTCGTTACAAATCATTAATTCTTTGTTTTAG**ACCCTTCGGAGGGAGAAGCAAGCCAATTCGCAGGATCTGGTTCAGTTGCAACATTTGCAACAGGATCTCGCACACAAGGAAAAGTGCCTGGCGGAAATGACCGCATTCGTTAATGCAGAGACCCAGCAGAAGAACGAGGTGATTCAGCAGCAGGCTCAGCAGCTGCAAGCCCTGGAGCTGCAACGCGAAGAGCTGGAAGCGCGCCAGAACAATAGCATCTTTGAGCTAGAGCAGCGCAAGCAGCTGGAGGCGGAGAACGCCGACCTCAAGCAGGAGCTGAGCGCTGTTCAACAGACTCAGTCGGAGCTGCAGCGCGTCCACGCCGCCGAGTTGCAGGAGCTGCGACAGAACTTGTCCGTCCTAGAGGCTCGCAACGTTGCGCTCAGCCAACAGCTCACCCAAGCCGCCAACAGCGCGGTTCAGGCCACCGCCGCCCAGTCGGAGCAGGCCCAGGTTCAGACCGAAGCGCTGGCCCAGAAGCAGCAGGAGCTGAGTGCTCTTCGCTCGCAGGTCGGCTCGCTGACGGATGCCCACGCCCAGCAGCAGAAGCAAGCCAACGCTCTGCAGTCGCAGCTCCAGGAGGCTCAACAGCGGGCCGAGCAGCTGCAGGCTAAGGAGCAACATCTGCAACAGGAGCTCCAGGAGCAGCGGGAGAAAAATAAT**GTGAGTATAGAGATGAACTCTATAACTAGAAATATATATTAAAAAACTTTTCTTTTTGTGTTGCATTCCCATTACATTCCCTATTTTATACCACCTACTTTAAACTTTATTGCACTTTACTTTGTAATCACTGAAATTGATACATATATATGTCAAGACTTATGCATGAAGATTAATAATGAATGCGGATAATTGAATTGAAAAGGATTTCAAACCAATTTGAGCCCAACTATATGTAATCGCGTCGCATTTGAGTCGTTATGCCGTCGTTCGTCCTGCCCGTTGCATCTGCATCCTTCTTGCTTTTCCTGAATCCTTTACTAATTGCCACGTATTCGTGTTTCGTCAG**GACGTGCGTATGAAAAATTGGAAGTTGATTGAAGCGTTGCAAAATGCCGAAGCATTAACAGCTAAGACGAAAACAAATTCAGCGCAGTCCGTAGGC**GTAAGTGCATGATCTTTGTCAGCTTCTTCTGCTTGCCTATATGATTCCATCTGAAAATTCGTAGAGTCTAAATCATTGTAAACTAATTGTATGCATTAATCTTTGCAG**CAACAACACAAAGAGCTGCAGCTGCAGCAACAGAAGGCAGTGGCCGCCAATGGCGGGGGTAGTGCCAGTTCAGCCAAG*DOC*AGCGAGCAGCAGAGGATCCGGGATCTCTACCAGCGCCTTTATCCCGACGCTGTGAAAGCGCAGTCGGGAAATGCCCTGCAAGCATCCTTTGACCAGTGGCTGGAACAGGTCCTGGCCACCCATGTCAAGCAGCAACAGGATAAGCTGCGGCAGAAGCTCGACGCGGAGAAGTCCGAGAAGCAGAGCAGCAGTAGTCATAAGTCCACACAATCAAGCAACAGTAGCAGTAGCAACCACAATAGCACCCACAACAATATTAGTAGCAATAATAGTAGTTCGAATAGCCAATCCTCCTCCGCCGCCGAGCAGCAGGAGCTGCACAAACAGAACCTGCAGCTGCGGGAGTGCAACGACAAGCTCACCCAGCTGGTCACCAAGACG**GTAATTACTTTTGCCCTATAGTAATTTATAACCTATCCAATGACAATAAAATCTTACGAACAG**ACAAACACATTGATGGACTTGGAAGAGCGTGCTCGTGAGCAGGACGAGCACTGGCGTGGCATCGTCGAGCAGAAGGAGCAGCTGATCCTAACCCTGCAGCAGCATGCCTCAAACGGAGAATAG**GTAAGGAATTGTGTTGCTGGTCTATAATAACTAGATTTCTAAACATATCGTTTGTCTTTTTGCAG**GACATTTAATGTCGAAACAGATGCGGAAAGGGAAGAGGAGGCCAACAACGACGATAAACTGACAAGCAACCGCAGAGTTTGTCCAACTTCCAAAAGAACAGCAACAGTAAAAAAGAAACAACTCTAACAATCTATTAAGTTTAAAGTTAATGTAACAAAAAAAAAAGGCGAGAAAAAAGTAAACCTAAAGCATGCCCCACAACAGCAACATTTAACATTTTATAGTAGCGAGAACCAACAATTATGAGAATGAAGAACTATGGAACGCTTTAAGAAATTATATACACATAACTAAAGCAAGATAATGATAACAATCGAAACTTGTGCAATGTTAAACGAGAAGAGAAGAAATGCTAGCAAAGGAAAAGTCCGACTCTGATTACAGAAACAGGCAATTTCCACCCAAGCCCTCCCAATAAAAAGCAAACCAACCCGGAAAAACCGCGAAAAATCCATTGCTGCTGCTACTTTCTAATTATTATTTAACACATACTTTGCATGCGATTATTATTTTTGTGTTACACTTGCATTTAAAAACGCGCTTTAAACAATTGTATATGTATCTTATGTTAAGCAAATGAATTTAAAACCCAGCTACTTAAAATGCAAATCCACAAAAAAATAAACAAAATAATAGTATGAATGTACGTATGCTCTTGTACTTGGGCCATTGTGAATTTTGTATGTTTTCCATTCATGTTTTCTGTTTTTGTGTTAACTTTTAATTTTCCGCCGCCAGGCAGCGCAAAGTATTTAATCCCAGGAAGAGCAAATGGCAGCAGCAACATTTAATTAAGCGTAAATAAACAAGAAAACAACTATTTTTATATAAAACTGAATGAGAACCTATCTTATAAGTAAACGCAAATTAAACACGAATGCTAACTAGCAATCTATTGTCGGAACCTGTTGCATGCGACATACTGAAAAATTAGGAAAACCAACAAATTCAAGCCCATCAGCAACCACAACAAACAAAAGAACGAGCTATAACAGCATTATAAGGATTTATGATGTTTAATTTACGATAAAATGAGAATTAAATATATACACACACATATGAGAAAGACATTTGACCTTTGACATTTCACT**

**Internally Deleted Hobo Insertion**

**In Same orientation as above:**

**TSD: GCCGCTGAA**

**Transcription Intitiation Sequences::CAAT/TATA**

**Putative Poly(A) Sequences (Upstream of deletion): AATAAA**

**5U and UGUGU (U and UG rich sequences): TTTTT/TGTGT (**RNA Recognition by the Human Polyadenylation Factor CstF)

**Hobo (1-926)**

**GAGAACTGCAAGGGTGGCACTTTTTTACCACTCGACTCACACCCTACAATTTTGTGTGCGGGTGCTACTCGCCACGCACATCGCGGGTACTTACAAACACACAGTATAAATCTGAACATGCAGACAAGACACCCCGTTGTGTGCGCACCCGAATCAATACGGTGTTTTGCGTCGCGGGTGCCGCTCACACAGTGCCTAAAAAGGGATGAGTGAGAAAAACACTTGTGGGTATACCGTTAAACACATGGGTGTTTCCAAAAATACTCGGGTGTTTCCAAAAATACTCGAGTGGTCTCGTAGGTAGTCGAGTCAAATGGCGCCATACATAATGATTGTTGAGTTCTTGTGTCTTTGGTCCAGTGTCTCGGCTGTTAATTGCCCCTTTTTTGTTTTTTACGATGCAATTACTAGCTTGTTAGGATTCAGTATTATTTGGAAGCCAAAGGAAAAGGTCACAATAATGGCAGAAGCGGCTGATTTCGTTAAAAATAAAATTAACAATGGAACATACTCAGTTGCCAATAAACATAAAGGAAAAAGTGTTATTTGGAGCATTTTATGTGACATTTTAAAGGAAGATGAAACTGTTCTGGACGGATGGCTGTTCTGCAGGCAATGCCAGAAAGTGCTCAAATTTTTACACAAAAACACCTCCAATTTATCCCGCCATAAATGTTGTCTAACATTAAGACGACCAACGGAATTAAAAATTGTTTCGGAAAACGACAAGAAAGTAGCTATTGAAAAATGCACCCAATGGGTTGTCCAAGATTGTCGGCCGTTTTCTGCAGTAACCGGAGCCGGATTTAAAAATTTGGTGAAGTTTTTCCTACAAATCGGCGCTATCTATGGGGAACAGGTAGACGTCGATGACTTACTACCTGATCCAACAACATTAAGTCGGAAGGCCAAATCGGATGCAGA**

**Hobo (2420-2959)**

**AGATGCAAAATCCTAAAAATGTGAAAGTAATGAAGTTCCTTATATTTAATAGATACTTTTTAAGCCCACTATGTTTTTATTATTTAGATTGAGACATTAAAAAACGTAAAAATCAACAAATGCCGTCTTTAATTGCAATTACTTTATGTGTTTGAAATGGGAGGCACCCATTGAGTCCATCAAAGAGCAAAGACATGAGCACAAAAATTTTCTTGGGTATTCCCTTTTACCCTTCATTTCTTATACCCGTCACGCTTCCACCCATACAAATTTTAGGCGTACAAAAAATGACCAGAGAACTGCAGCCCGCATACAAAAAATGACCTGCGGCCGATCGTTGACTGTGCGTCCACTCACCCATACGGCTCTTGCGCAGCAGGCCTCGGGTGGTTTTTTTACTCGTAACAAAAACACAACGTCGGTAAAACACTCGAGTATTTTGTGTTGCCGCAAGTAGGGTGTCAAAAAAAACGGGGTGCCTAGAGTACCGAGTGTTTATCGGGTGGACGTAGAGTGCGAGTGGCGGGCTGCAGTTCTCTG**

**Density of UGUGU and UUUUU downstream of last AAUAAA sequence in Hobo: 7/798=0.0075**

**Density of UGUGU and UUUUU in full preceding sequence: 33/8784=0.0038**

**Truncated Doc Insertion (Not fully sequenced. Partially Inferred)**

**In same orientation as above**

**CGAACTGACCGAACAACCCAACGAAAAAACGGTAGGGGAACCAAAAAAGACCAGGCCTCCACCAATTTTCATACGAGAACAAAGTACAAATGCACTTGTAAATAAACTCGTTGCTTTGATTGGTGACAGCAAATTGTACATTATCCCACTTAAAAAAGGAAATATTCATGAAATAAAACTACAGATCCAAACAGAAGCAGACCACCGTATAGTGACTAAATACCTAAATGATGCTGGTAAAAACTACTACACATACCAATTAAAAAGTTGCAAAGGGCTACAGGTAGTACTTAAGGGCATTGAAGCAACAGTGACACCAGCTGAGATAATTGAGGCTCTGAAGGCCAAAAACTTTTCTGCAAAGACAGCTATTAATATTTTAAACAAAGACAAAGTTCCGCAGCCACTATTCAAAATAGAACTCGAACCAGAGCTCCAGGCACTAAAGAAAAACGAAGTGCACCCAATATACAATTTACAGTACTTGCTACATCGGAGGATCACCGTGGAGGAGCCGCACAAACGTATCAATCCAGTTCAATGTACTAATTGCCAAGAATACGGCCACACCAAGGCATACTGCACCCTTAAGTCCGTATGTGTTGTCTGTAGCGAACCTCATACTACCGCAAACTGCCCCAAAAACAAGGACGATAAGTCTGTGAAGAAATGCAGTAACTGCGGGGAAAAACATACTGCAAACTACAGAGGCTGTGTGGTGTACAAAGAATTGAAGAGCCGCCTAAACAAACGTATTGCCACAGCACATACATACAACAAAGTCAATTTCTACTCTCCGCAACCGATTTTTCAACCACCCCTAACTGTCCCAAGCACTACTCCAACAATTTCTTTCGCTAGCGCCCTAAAATCCGGACTAGAAGTGCCCGCCCCACCGACAAGAACTGCTCATTCCGAACATACACCGACAAACATCCAACAAACACAACAAAGTGGCATCGAAGCTATGATGCTATCCCTACAGCAAAGCATGAAAGACTTCATGACGTTCATGCAAAATACTTTGCAAGAGCTCATGAAAAACCAAAATATCCTGATTCAACTTCTTGTATCTTCAAAATCCCCATAATGGCTTCCCTACGGATATCTCTGTGGAACGCAAATGGCGTTTCACGGCATACACAAGAGCTCACACAGTTCATTTACGAAAAAAACATCGACGTAATGCTACTATCAGAAACGCACCTCACAAATAAAAACAATTTTCATATACCAGGATACTTGTTCTATGGTACAAATCATCCAGATGGTAAAGCTCATGGAGGCACTGGAATACTCATCAGAAATCGCATAAAACACCACCACTTAAACAATTTTGACAAAAACTACTTACAATCTACGTCCATAGCCTTACAACTCAACAATGGTTCAACGACTCTAGCCGCAGTCTACTGCCCACCGCGCTTTCCAATCTCTGAGGATCAATTCATGGAATTCTTTAACACACTAGGTGACAGGTTCATCGCAGCGGGTGACTATAACGCCAAGCACACCCATTGGGGATCTCGACTTGTGTCGCCAAAGGGTAAGCAATTGTACAATGCGCTTACGAAGCCAGAAAACAAGCTAGACTATGTATCCCCGGGTAAGCCTACATACTGGCCAGCAGACCCAAGAAAAATCCCAGACCTGATCGATTTTGCAATTACTAAACATGTCCCCCGCAACATGGTCACCGCCGAAGCACTAGCAGATTTATCATCAGATCACTCACCTGTTTTTCTAAATATGCTAACTCGCCCCCACATCGTCGACCCACCGTATAGACTCACAAATTTTAGAACAAACTGGCCAAGGTATCAAAAGTATGTCTGTTCACACATAGAACTAACGACGGCATTATCTACAAAGGAGGATATAGACAAGTCAACGGAAACTCTTGAAAACATTTTAGTTTCGGCTGCAAAGGCTTCAACCCCGCCAGTGACGTATGCAAAACCAAACTACATCAAAACTAATCGCGAAATCGAGCGGCTGGTATTAGATAAACGACGCCTACGAAGGGATTGGCAGTCTAATAGATCACCAATTACTAAGCACATGCTTAAGATAGCCACACGCAGGCTTACCAATGCTCTCAAACAAGAGGAAAAAAACAGCCAACGTTCATATATCGAGCAACTCTCTCCCACCAGCACTAAGTACCCTCTTTGGAGAGCTCACAGAAACCTAAAGACTCCAATAGCGCCAATTATGCCACTCCGAAGTCCCTCTGGCACCTGGTTTCGAAGTGATGAAGAAAGAGCCAGTGCTTTCGCTGACCATTTACAAAATGTATTCCGACCAAATCCCTCTACCAACACATTTATTCTCCCTCCTTTAATAGCAGCCAATCTAGATCCTCAAGAACCCTTTGAATTCCGACCATGTGAACTAGCAAAGGTTATCAAAGAGCAACTGAACCCAAGAAAATCGCCTGGCTACGACCTAATAACTCCAAGAATGCTCATTGAACTCCCAAAGTGTGCTATTCTTCACATCTGCCTGTTGTTCAACGCAATCGCCAAGCTTGGATACTTCCCTCAAAAATGGAAAAAGTCGACCATAGTAATGATTCCAAAGCCAGGAAAAGATAAAACGCAGCCATCATCATATAGACCGATAAGCTTACTAACATGTCTTTCAAAGCTGTTTGAAAAAATGCTACTCCTTCGGATTAGCCCTCATCTTAGAATAAACAACACACTTCCAACACATCAATTTGGCTTTAGAGAAAAACATGGAACCATCGAACAGGTCAACCGAATCACGTCAGAAATTCGTACTGCTTTTGAACATCGAGAATACTGCACAGCCATTTTTCTAGACGTCGCGCAGGCATTTGACAGAGTGTGGCTCGATGGACTTTTGTTTAAAATAATCAAGCTGTTGCCCCAAAACACACATAAGCTACTGAAGTCATACCTATATAACAGAGTGTTTGCAATAAGATGCGATACAAGCACTTCACGCGATTGCGCAATCGAAGCTGGAGTGCCGCAAGGCAGTGTACTGGGTCCAATCTTATACACCCTGTATACGGCGGATTTCCCCATAGACTACAATCTAACAACCTCCACGTTCGCTGATGATACCGCGATACTCAGTCGCTCGAAATGCCCAATAAAAGCCACGGCACTCCTATCCCGACACTTAACATCTGTAGAACGATGGCTTGCCGACTGGAGAATTTCAATAAATGTTCAAAAATGCAAGCAGGTTACCTTTACCTTAAACAAACAAACATGCCCACCACTGGTCTTGAATAACATATGCATTCCACAAGCCGACGAGGTAACATATCTGGGAGTTCATCTGGACAGGCGGCTCACTTGGCGCAAACATATAGAAGCCAAATCGAAACATCTTAAACTTAAAGCAAGGAACCTCCACTGGCTCATAAATGCTCGCTCTCCACTTAGTCTGGAGTTCAAAGCTCTTCTATACAACTCCGTCTTAAAACCTATCTGGACTTATGGCTCCGAGCTGTGGGGCAACGCATCCAGAAGTAACATAGACATTATTCAGCGAGCACAGTCAAGAATTCTGAGAATTATCACTGGAGCGCCGTGGTACCTTCGAAACGAAAACATACACAGAGACCTAAAAATCAAATTAGTAATCGAAGTAATAGCTGAGAAAAAAACGAAGTATAACGAAAAGCTGACCACCCATACAAATCCCCTCGCAAGAAAACTAATCCGAGTATGCAGTCAAAGCCGGCTGCACCGCAACGACCTCCCAGCCCAGCAATAAACTTATTAGGGCATTAATGAAAAAAAAAAACTATCACTAAGTGAAAGTTAATTAAGTTAGATTAAGATTTGAACACTTATTGTTAGTCTCTTAACACAAAGGGAAGATTCAATAAATAATAAAAATTAAAAAAAAAAAAAAAAAAAAAA**
