# Supplementary material for: Off-target piRNA gene silencing in Drosophila melanogaster rescued by a transposable element insertion
Source: PLoS Genet. 2023 Feb 21;19(2):e1010598. doi: 10.1371/journal.pgen.1010598 (PMC9983838; doi:10.1371/journal.pgen.1010598)
Supplement: S2 File — (PDF) [file pgen.1010598.s006.pdf]

# <u>Oligo List</u>

**F18nt Control**
*(18-nt custom RNA oligo from firefly luciferase CDS) – (Zamore)*
5′-GGUGGACAUCACUUACGC-3′

**2S Block**
*(30-nt custom DNA oligo with the 3' 3SpC3, HPLC purified) – (Zamore)*
5′-TACAACCCTCAACCATATGTAGTCCAAGCA/3SpC3/-3′

**3' piRNA Adapter**
*(31-nt custom DNA oligo with* **NNNNNN** *used as a molecular identifier) – (Blumenstiel)*
5′-/rApp/**NNNNNN**AGATCGGAAGAGCACACGTCTGAA/ddC/-3′

**RT primer # (# = 01-12)**
*(***XXXXXX** *is a unique Index used to distinguish samples at sequencing) – (Blumenstiel)*
5′-CGGCATACGAGATAGTCCAG**XXXXXX**GTGACTGGAGTTCAGACGTGTGCTCTTCCGATCT-3′

| Sequence of Index in Primer | Reverse Compliment of Index |
|---|---|
| Index 01 – CGTGAT | Index 01 – ATCACG |
| Index 02 – ACATCG | Index 02 – CGATGT |
| Index 03 – GCCTAA | Index 03 – TTAGGC |
| Index 04 – TGGTCA | Index 04 – TGACCA |
| Index 05 – CACTGT | Index 05 – ACAGTG |
| Index 06 – ATTGGC | Index 06 – GCCAAT |
| Index 07 – GATCTG | Index 07 – CAGATC |
| Index 08 – TCAAGT | Index 08 – ACTTGA |
| Index 09 – CTGATC | Index 09 – GATCAG |
| Index 10 – AAGCTA | Index 10 – TAGCTT |
| Index 11 – GTAGCC | Index 11 – GGCTAC |
| Index 12 – TACAAG | Index 12 – CTTGTA |

**5' SR Adaptor**
*(BA5, 26-nt custom RNA oligo, PAGE purified) – (Zamore)*
5′-GUUCAGAGUUCUACAGUCCGACGAUC-3′

**PCR Forward Primer**
*(BPCRP1. 50-nt custom DNA oligo) – (Zamore)*
5′-AATGATACGGCGACCACCGAGATCTACACGTTCAGAGTTCTACAGTCCGA-3′

**PCR Reverse Primer**
*(31-nt custom DNA oligo) – (Blumenstiel)*
5′-CAAGCAGAAGACGGCATACGAGATAGTCCAG-3′

**110-nt Oligo**
*(110-nt custom DNA oligo, used as lower band for ladder on polyacrylamide gel)*
5′GATGCTTAGGATCCCATGCATGCTAGCTTTACGGAACCTACGGACGTTAAGCCCACAGAGATATGCAC
CTGGACTTCAAGGGCTGATCGGATCGGATCGGATCGTAGATC-3′

**124-nt Oligo**
*(124-nt custom DNA oligo, used as upper band for ladder on polyacrylamide gel)*
5′GATGCTTAGGATCCCATGCATGCTAGCTTTACGGAACCTACGGACGTTAAGCCCACAGAGATATGCAC
CTGGACTTCAAGGGCTGATCGGATCGGATCGGATCGTAGATCGTGTCATTAGCTCG-3′

**110nt RT Product Mimic**
*(110-nt custom DNA oligo that is a replicate of our desired RT product on an* ***18bp fragment.****)*
5′CGGCATACGAGATAGTCCATCGTGATGTGACTGGAGTTCAGACGTGTGCTCGGCCGATCTGGACTC**GG**
**TGGACATCACTTACGC**GATCGTCGGACTGTAGAACTCTGAAC3′

**122nt RT Product Mimic**
*(122-nt custom DNA oligo that is a replicate of our desired RT Product on a* ***30bp fragment.****)*
5′CGGCATACGAGATAGTCCATCGTGATGTGACTGGAGTTCAGACGTGTGCTCGGCCGATCTGGACTC**AG**
**TATGGGCATTTCGCAGCCTACCGTGGTG**GATCGTCGGACTGTAGAACTCTGAAC3′

# <u>Solutions List</u>

**Nuclease-free water – Super $H_2O$**
Sterile and nuclease-free; used for everything in RNA world and for most solution prep.

**Milli-Q water**
Used for some solution prep and gel set-up. Never when dealing with RNA.

**RDS**
Homemade RNase-away is made by diluting 50 mL of 1M NaOH and 1 mL of 0.5 M EDTA into 449 mL of nuclease-free water, for a final volume of 500 mL. Final concentrations are 0.1 M NaOH, 1 mM EDTA.

**1 M NaOH - Sodium Hydroxide**
Dissolve 20.0 g of NaOH (Fisher, S318-1) into 400 mL of nuclease-free water. Add water to bring final volume to 500 mL.

**0.5 M EDTA - Ethylenediamenetetraacetic acid**
Dissolve 93.06 g of EDTA (Fisher, S311500) into 400 mL of nuclease-free water. Add water to bring final volume to 500 mL.

**0.1% DEPC Water**
Dilute 800 µL DEPC (Sigma, D5758) (stored at 4 degrees, thaw to room temp before using) in 800 mL Milli-Q water. Shake and let sit overnight in the hood with caps loosely secured so bottle can vent. Autoclave.

**TRI Reagent**
RNA extraction performed in Trizol (Ambion, AM9738).

**Ethanol**
(BioStore) Need 4 ethanol concentrations: 70%, 75%, 80%, and 100%.

**2X Borate buffer (pH 8.6)**
8.76 mM Borax ($Na_2B_4O_7$, Sigma, 221732-100g) and 100 mM Boric acid ($H_3BO_3$, Sigma, B6768-500g). Dissolve 0.6183 g of Boric acid and 0.1763 of Borax into 80 mL of nuclease-free water. Add water to bring final volume to 100 mL.

**200 mM $NaOI_4$ - Sodium Periodate**
Dissolve 0.2139 g Sodium Periodate (Sigma, S1878-25g) in 5 mL water. Prepare just before use!

**Glycerol**
Glycerol (Sigma, G5516-500ML) does not need to be diluted.

**3 M NaOAc - Sodium Acetate**
Dissolve 40.8 g of NaOAc (Sigma, S2889-250G) into 70 mL of nuclease-free water. Adjust pH to 5.2 and add water to bring final volume to 100 mL.

**Glycogen**
Gylcogen (Roche, 10901393001) comes as 20 mg/mL stock and does not need to be diluted.

**10X T4 RNA Ligase Reaction Buffer**
The 10X 3' Ligation Buffer comes with T4 RNA Ligase 2, truncated KQ (NEB, M0373L) and contains 500 mM Tris-HCl (pH 7.5), 100 mM $MgCl_2$, 10 mM DTT.

**50% PEG 8000**
Comes with T4 RNA Ligase 2, truncated KQ (NEB, M0373L) and is used in the 3' ligation reaction.

**T4 RNA Ligase 2, truncated KQ**
The 3'Ligation Enzyme (NEB, M0373L) comes in 200,000 U/mL stock. This tube is contains 10,000 units. It is 50 µL of 200 U/µL, good for 33 samples.

**10X T4 RNA Ligase Reaction Buffer**
The 10X 5' Ligation Buffer comes with T4 RNA Ligase 1 (NEB, M0204S) and contains 500 mM Tris-HCl (pH 7.5), 100 mM $MgCl_2$, 10 mM DTT.

**10 mM ATP**
Comes with T4 RNA Ligase 1 (NEB, M0204S) and used in the 5' ligation reaction.

**T4 RNA Ligase 1**
The 5' Ligation Enzyme (NEB, M0204S) comes in 10,000 U/mL stock. This tube contains 1,000 units. It is 100 µL of 10 U/µL, good for 50 samples.

**5X ProtoScript® II Reverse Transcriptase Reaction Buffer**
The 5X RT Buffer comes with the ProtoScript® II Reverse Transcriptase (NEB, M0368X) and contains 250 mM Tris-HCl (pH 8.3), 375 mM KCl, 15 mM $MgCl_2$.

**100 mM DTT**
Comes with ProtoScript® II Reverse Transcriptase Reaction Buffer (NEB,M0368X) and is used in the RT reaction.

**10 mM dNTPs**
No dilution necessary (Qiagen, 201900), comes as 10 mM stock.

**ProtoScript® II Reverse Transcriptase**
The RT Enzyme (NEB, M0368X) comes in 200,000 U/mL stock. Formerly called M-MuLV Reverse Transcriptase (RNase $H^-$). This tube contains 40,000 units. It is 200 µL of 200 U/µL, good for 36 samples.

**Murine RNase Inhibitor**
RNase Inhibitor (NEB, M0314L) comes in 40,000 U/mL stock. This tube contains 15,000 units. It is 375 µL of 40 U/µL, good for 136 samples.

**SequaGel - UreaGel System**
The polyacrylamide gel kit SequaGel (National Diagnostics, EC-833) contains UreaGel Buffer, UreaGel Concentrate, and UreaGel Diluent

**10% ammonium persulfate - APS**
Dissolve 4 g of APS (Sigma, A3678-25G) in 40 mL nuclease-free water in a sterile 50mL falcon tube. Aliquot, and store in -20. Be careful making this, this needs to be RNase free.

**TEMED**
TEMED (Sigma, T9281) does not need to be diluted.

**0.5X TBE**
Prepare 2.5 L by diluting 125 mL of 10X TBE with 2375 mL of Milli-Q water.

**2X Formamide Loading Dye**
A 2X Gel Loading Buffer (Ambion, 8547) for denaturing PAGE. Contains 95% Formamide, 18 mM EDTA, and 0.025% SDS, Xylene Cyanol, and Bromophenol Blue.

**SYBR Gold**
SYBR Gold (Invitrogen, S11494) is supplied as 10,000X concentrate in DMSO: no dilution necessary,

**0.3 M Sodium Chloride - NaCl**
Dissolve 87.8 g of NaCl (Fisher, BP358-1) into 400 mL of nuclease-free water. Add water to bring final volume to 500 mL.

**LongAmp Taq DNA Polymerase**
The PCR enzyme (NEB, M0323S) comes as 2,500 U/mL stock: no dilution necessary.

**5X LongAmp Taq Reaction Buffer**
The 5X PCR Taq buffer (NEB, M0323S) contains 300 mM Tris-$SO_4$ (pH 9.1), 100 mM $(NH_4)_2SO_4$, 10 mM $MgSO_4$, 15% glycerol, 0.3% IGEPAL CA-630, 0.25% Tween 20

**5X Loading Dye**
From the Qiagen MinElute Kits.

**Agarose**

**1X TAE**

**Ethidium Bromide**

**25 bp Invitrogen ladder**
(Invitrogen, 10597-011)

**Isopropanol**

**EB buffer**
This elution buffer (Qiagen, 19086) contains 10 mM Tris-HCl (pH 8.5).

# Kit List

**Zymo Direct-zol Microprep Kit**
RNA extraction kit (Zymo, R2060) contains RNA Wash Buffer, Zymo-Spin IC columns, DNase I, DNA Digestion Buffer, Direct-zol RNA PreWash, DNase/RNase-Free Water.

**Qubit BR RNA Kit**
Quantification of RNA using this Qubit kit that contains, RNA BR Reagent, RNA BR Buffer, Standard 1 and Standard 2.

**Zymo Oligo Clean & Concentrator kit**
DNA concentrator kit (Zymo, D4060) contains Oligo Binding Buffer, Zymo-Spin Columns, Collection Tubes, DNA Wash Buffer, and DNase/RNase-Free Water.

**QIAGEN MinElute Gel Extraction Kit**
Gel extraction kit (Qiagen, 28604) that contains MinElute Columns, Collection Tubes, Buffer QG, Buffer PE, Buffer EB

**Qubit BR dsDNA Kit**
Quantification of DNA using this Qubit kit that contains, dsDNA BR Reagent, dsDNA BR Buffer, Standard 1 and Standard 2.

## Equipment List

Kimwipes
Ice
Dry Ice
500 mL glass beaker
Liquid Nitrogen
25mL disposable pipette
Nuclease-free Pestles
Nuclease-free 0.2 mL PCR tubes
Nuclease-free 1.5 mL microcentrifuge tubes
Costar Spin-X Filters (Sigma, CLS8160)
15 mL Falcon tubes
50 mL Falcon tubes
New Razor Blades
Centrifuge
Qubit 2.0
Thermocycler
Polyacrylamide Gel Casting Equipment
Plastic Staining Dish For Polyacrylamide gel
Aluminum Foil
Gel Shaker
Blue Filter Box
Bio-imager
UV light box
Amber goggles
Falcon Tube Shaker

# Library prep for sequencing piRNA

*Aim to collect at least* **1.5 µg** *of Total RNA.*

## Day 1 – RNA extraction, quantification and oxidation

✪*Make sure equipment is RNase free, spray with RDS, dry with Kimwipes, and rinse with DEPC water.*✪

### Step 1 - RNA Isolation via Zymo Direct-zol Microprep

1. Sample Preparation:

   1.1 Lyse tissue (≤ 5 mg) sample in 300 µL of TRI Reagent and homogenize with an RNase free squisher single.

   1.2 To remove particulate debris from homogenized tissue, centrifuge and transfer the supernatant into an RNase-free tube.

2. Add 300 µL EtOH (95-100%) to supernatant and mix thoroughly by inversion.

3. Transfer the mixture into a *Zymo-Spin IC column* inside a collection tube and centrifuge at 13,000×g for 30 seconds. Discard the flow-through.

4. DNase treatment in column:

   4.1 Add 400 µL *RNA Wash Buffer* to the column and centrifuge at 13,000×g for 30 seconds.

   4.2 In an RNase-free tube, add 5 µL *DNase I* (6U/µL), 35 µL *DNA Digestion Buffer* and mix. Add the mix directly to the column matrix.

   4.3 Incubate at room temperature (20-30°C) for 15 minutes.

5. Add 400 µL *Direct-zol RNA PreWash* to the column and centrifuge at 13,000×g for 30 seconds. Discard the flow-through and **REPEAT THIS STEP**.

6. Add 700 µL *RNA Wash Buffer* to the column and centrifuge at 13,000×g for 2 minutes to ensure complete removal of the wash buffer.

7. Discard collection tube and transfer the column carefully into a 1.5 mL RNase-free tube.

8. To elute RNA, add 7 µL* of *DNase/RNase-Free Water* directly to the column matrix and centrifuge.

   **Can elute in minimum of 6 µL, but a 7 µL elution is preferred because 1 µL is lost on column.*

### Step 2 – RNA Quantification via Qubit 2.0

9. Dilute 1 µL of product into 4 µL of nuclease-free $H_2O$ in 0.2 mL RNase-free tubes and use for quantification of RNA diluted 1:5 on the Qubit 2.0.

9.1 Set up the appropriate amount of 0.5 mL tubes for the samples plus the 2 Qubit standards.

9.2 Prepare the Qubit working solution in a 15 mL Falcon tube by diluting the Qubit RNA BR Reagent 1:200 in Qubit RNA BR Buffer.

9.3 Add 190 µL of Qubit working solution to each of the tubes used for standards.

9.4 Add 10 µL of each Qubit standard to the appropriate tube.

9.5 Add 199 µL of Qubit working solution to each of the tubes used for the samples.

9.6 Add 1 µL of each sample to the appropriate tube.
NOTE: The final volume of every tube is now 200 µL.

9.7 Vortex every tube for 2-3 seconds, being careful not to create bubbles.

9.8 Allow all tubes to sit at room temperature for 2 minutes.

9.9 Read Standard 1, followed by Standard 2, to calibrate the instrument.

9.10 Read each sample to get the Assay Tube Conc ($\alpha$). Then use the Qubit to calculate the Stock Conc ($\beta$). It is important to remember that Stock Conc is a 1:5 dilution of the sample. So Sample Conc ($\omega$) must be calculated.

10. Dilute all samples to the same concentration with nuclease-free $H_2O$.

11. Place 0.5-1.0 µg of sample into a 1.5 mL PCR tube and if necessary add nuclease-free $H_2O$ to bring the final volume of 8.75 µL. Label it as the non-oxidized (-) sample.

12. Place another 0.5-1.0 µg of sample into a separate 1.5 mL PCR tube and if necessary add nuclease-free $H_2O$ to final volume of 8.75 µL. Label this tube as the oxidized (+) sample.

**NOTE: Freeze the remaining amount of total RNA at -80°C. This will be used later for RNA-seq via the Illumina kit.**

## Step 3 – piRNA Enrichment via Sodium Periodate Oxidation

13. Perform the **piRNA Enrichment via Oxidation** protocol.

13.1 Add 8.75 µL of 2X Borate Buffer to all samples.

13.2 Oxidized Samples: Add 2.5 µL of 200 mM $NaIO_4$.
Non-oxidized Samples: Add 2.5 µL of nuclease-free water.

13.3 Incubate the reaction for 10 min in the dark at 24°C.

13.4 Quench the reaction by adding 1 µL glycerol (5% final) and incubate for an additional 10 min in the dark at 24°C.

13.5 Add 248 µL of water, 30 µL of 3 M Sodium acetate (pH 5.2), and 1 µL of glycogen (20 µg) to each tube and vortex briefly to mix and then spin down.

13.6 Add 3 volumes (900 µL) of 100% EtOH to each tube and vortex briefly to mix. **Incubate samples on ice for 1hr.**

13.7 Spin at 17,000×g for 15 min at 4°C. Then vortex for 10 seconds to strip precipitates from the wall.

13.8 Spin at 17,000×g for 15 min at 4°C. Then gently remove and discard the supernatant without touching the pellet.

13.9 Add 900 µL of 75% EtOH to each tube and vortex briefly.

13.10 Spin at 17,000×g for 5 min at 4°C. Then gently remove supernatant without touching the pellet.

13.11 Spin at 17,000×g for 1 min at 4°C to pull down residual ethanol. Discard any ethanol with p10 pipette.

13.12 Air dry for 2 min then dissolve the pellet with 2 µL of nuclease-free water.

13.13 Transfer all 2 µL samples, oxidized and non-oxidized, to 0.2 mL PCR tubes.

*NOTE: RNAs containing vicinal 2' and 3' diol groups react with $NaIO_4$. Periodate treatment therefore selectively enriches for piRNAs since they are 2'-O-methylated and thus resistant to periodate treatment, while miRNAs contain 2' and 3' terminal hydroxyl groups and are reactive and altered to prevent 3' adaptor ligation during library preparation.*

14. If stopping, place all 2 µL samples, oxidized and non-oxidized, in the -80°C freezer. If continuing, proceed with all samples to **2S Block.**

**✪★SAFE STOPPING POINT ★✪**
**If stopping, place samples in -80°C.**

## Day 2 – 2S Block and 3' Adapter Ligation

*✪Make sure equipment is RNase free, spray with RDS, dry with Kimwipes, rinse with DEPC water. ✪*

### Step 4 – 2S Block

15. Start the **18nt Control:** Dilute the 18-nt RNA oligo, with super water, to 0.5 nmol/µL in a RNase free 1.5mL microcentrifuge tube. Place 2 µL* of the diluted 18-nt RNA oligo in a 0.2 mL PCR tube. Perform remainder of protocol on this sample. It will be used as a control for the final gel size-selection step.

**2 µL of diluted 18-nt RNA oligo is equivalent to 1 nanomole; which about 100x the estimated total amount of target RNA in the sample*

16. Add the 2S Block to each sample in their sterile, nuclease-free 0.2 mL PCR tube:

| *Starting Components* | *Volume (µL)* | *Final Conc* |
|---|---|---|
| 0.5-1.0 µg* of Input RNA | 2 µL | |
| **100 µM 2S Block** | 1 µL | 100 pmoles** |

| | |
|---|---|
| Total Volume | 3 µL |

**If using <100ng of total RNA as input, dilute the 3' piRNA Adaptor, RT primers and 5' SR adaptor 1:2*
***This is ~100 fold excess of 2S RNA (if 2S = 1% total RNA)*

17. Incubate in a preheated thermal cycler for 2 min at 70°C. Transfer to ice.

**Step 5 – Ligation of the 3' piRNA Adapter**

18. Add the following components to each sample:

| *3' Adapter Ligation Components* | *Volume (µL)* | *Final Conc* |
|---|---|---|
| 10X T4 RNA Ligase Reaction Buffer | 0.700 µL | 1 X |
| 200 U/µL T4 RNA Ligase 2, truncated KQ | 0.875 µL | 25 U/µL |
| 50% PEG 8000 | 1.425 µL | 10.17% |
| **10 µM 3' piRNA Adaptor** | 1.00 µL | 10 pmoles |
| Total Volume Added | 4.0 µL | |
| Previous Volume of Sample | 3.0 µL | |
| Final Volume of Sample | 7.0 µL | |

19. For targeting methylated piRNAs: Incubate for 18 hrs. at 16°C. (For all other small RNAs: Incubate 1 hr. at 25°C).

**✪★SAFE STOPPING POINT ★✪**
**Samples must incubate at 16°C for 18 hrs.**

## Day 3 - Annealing the RTPs, 5' Adapter Ligation, Reverse Transcription and Hydrolysis

✪ *Make sure equipment is RNase free, spray with RDS, dry with Kimwipes, rinse with DEPC water.* ✪

**Step 6 – Annealing the Reverse Transcription Primers (RTPs)**

20. Remove sample from incubation and place on ice.

21. Add the unique RT Primer directly to each sample.

| *RT Primer Ligation Components* | *Volume (µL)* | *Final Conc* |
|---|---|---|
| **100 µM RT Primer*** | 1.0 µL | 100 picomoles |
| Total Volume Added | 1.0 µL | |
| Previous Volume of Sample | 7.0 µL | |
| Final Volume of Sample | 8.0 µL | |

**Each sample has its own unique RT primer. 100 picomoles is 10X more RT primer than the 3' adapter.*

22. Incubate in thermocycler: 1 min at 90°C, then 5 min at 65°C, then 15 min ramp down to 4°C.

**Step 7 – Ligation of the 5' SR Adapter**

23. Aliquot 1.1*N µL of the 5' SR Adaptor into a separate nuclease-free 200 µL PCR tube, with N equal to the number of samples being processed.

*If SR adapter has already been diluted 1:2 and stored at -80°C, aliquot 2.1 N µL instead of 1.1 N.

24. Incubate the adapter in the thermal cycler for 2 min at 70°C and then immediately place the tube on ice. Keep the tube on ice and use the denatured adaptor within 30 minutes of denaturing.

**NOTE: Store remaining resuspended 5' SR Adapter at -80°C.**

25. Prepare a mastermix with the following components in a 1.5 mL RNase-free tube, then add 4 µL to each sample:

| *5' Adaptor Ligation Components* | *Volume (µL)* | *Final Conc* |
|---|---|---|
| Nuclease-free water | 0.1 µL | |
| 10 mM ATP | 1.2 µL | 1 mM |
| 10X T4 RNA Ligase Reaction Buffer | 0.5 µL* | 1 X |
| **10 µM 5' SR Adapter (denatured)** | 1.0 µL** | 10 picomoles |
| 10 U/µL T4 RNA Ligase 1 | 1.2 µL | 1 U/µL |
| Total Volume Added | 4.0 µL | |
| Previous Volume of Sample | 8.0 µL | |
| Final Volume of Sample | | 12.0 µL |

*0.7 µL of 10X T4 RNA Ligase Reaction Buffer already present, so only 0.5 µL needed for 1X final concentration.

**If SR adapter has already been diluted 1:2 and stored at -80°C, add 2 µL instead of 1 µL and only 1 µL of water.

26. In a thermal cycler, incubate for 1 hr. at 25°C.

## Step 8 – Reverse Transcription and Hydrolysis

27. Prepare a mastermix with the following components in a 1.5 mL RNase-free tube, then add 13 µL to each sample:

| *Reverse Transcription Components* | *Volume (µL)* | *Final Conc* |
|---|---|---|
| 5X Protoscript II Reverse Transcriptase Reaction Buffer | 5.0 µL | 1 X |
| 40 U/µL Murine RNase Inhibitor | 0.5 µL | 0.8 U/µL |
| 10 mM dNTPs | 2.5 µL | 1 mM |
| 100 mM DTT | 2.5 µL | 10 mM |
| 200 U/µL Protoscript II Reverse Transcriptase | 2.5 µL | 20 U/µL |
| Total Volume Added | 13.0 µL | |
| Previous Volume of Sample | 12.0 µL | |
| Final Volume of Sample | | 25.0 µL |

28. Incubate for 1 hr. at 42°C.

29. To hydrolyze remaining RNA in the samples after RT, add 5 µL of 0.5 M EDTA and 5 µL of 1 M NaOH to every sample.

30. Incubate sample for 15 min at 65°C.

31. Pool 30 µL from each oxidized sample together and 30 µL from each non-oxidized sample together in separate 1.5 mL tubes (max 50 samples/tube).

**NOTE: The remaining 5 µL of each sample will be saved and stored in the -20°C to be used as individual controls if needed.**

**✪★ SAFE STOPPING POINT ★✪**

**Continue to Oligo Clean & Concentrator kit or store in -20°C.**

## Day 4 – Concentrate cDNA and PAGE gel

✪ *If planning to run polyacrylamide gel in the same day, set up gel (step 40-45).* ✪

### Step 9 - Concentrate cDNA via Zymo Oligo Clean & Concentrator kit Protocol

32. Split each pool into 100 µL aliquots in 1.5 mL microcentrifuge tubes and add 200 µL *Oligo Binding Buffer* to each one.

33. Add 800 µL of ethanol (95-100%) to each aliquot, bringing the total volume to 1100 µL/tube and mix briefly by pipetting.

34. Transfer 550 µL to a *Zymo-Spin*™ *Column* in a *Collection Tube* and centrifuge at 13,000×g for 30 seconds. Discard the flow-through.

35. **REPEAT step 34 until the entire pool has been passed through its own spin column.**

36. Add 750 µL *DNA Wash Buffer* to the column. Centrifuge at 13,000×g for 30 seconds and discard the flow-through. Then centrifuge at maximum speed for 1 minute to remove residual ethanol.

37. Discard collection tube and transfer the column carefully into a 1.5 mL RNase-free tube.

38. To elute DNA, add 11 µL* of *DNase/RNase-Free Water* directly to the column matrix and let stand for 3-5 minutes.
    *Can elute with a minimum of 6 µL. Zymo states 1 µL is lost on the column during elution.

39. Centrifuge at 13,000×g for 30 seconds to elute DNA. If gel is set up already, continue to step 46 in **10% Polyacrylamide gel separation**. If gel wasn't made yet, freeze samples in -20°C.

**✪★SAFE STOPPING POINT ★✪**
**If stopping, place samples in -20°C. Ideally, continue to PAGE gel, if time allows it.**

### Step 10 - 10% Polyacrylamide gel separation of small RNA (now cDNA)

✪*Must wear lab coat and gloves when working with UreaGel components* ✪
✪*Liquid nitrogen is needed for flash freeze step at step 55*✪

40. Clean the glass plates and spacers to be used for 1.5mm gel with 70% EtOH. Prepare an ice bucket to be used for making the gel solution and thaw frozen 10% APS (ammonium persulfate).

41. Setup "sandwich" on casting stand.

    41.1 Larger glass plate down first, then white spacers – the spacers will snap together on bottom, make sure they're snug.

    41.2 Align the orange gaskets so they are flush with the top of the larger plate.

    41.3 Place the short plate on top, flush with the bottom and sides.

41.4 Place your hand in the center of the glass plates to hold them steady and place clips around the plates to secure. The bottom clips can be folded out so they act as a stand.

42. Place a 500 mL autoclaved glass beaker on the ice and add all UreaGel components (using a serological pipette and a pipette aid). Keep on ice to prevent early polymerization.

| *Component* | *Volume (mL)* |
|---|---|
| UreaGel Concentrate | 20.0 mL |
| UreaGel Diluent | 25.0 mL |
| UreaGel Buffer | 2.5 mL* |
| Milli-Q water | 2.5 mL |
| Total Volume | 50.0 mL |

* Using half the recommended amount of UreaGel Buffer because the gel is run in 0.5X TBE instead of 1X.

43. Mix solution by swirling beaker and return to ice. Add the following components and mix by swirling after each one is added:

| *Component* | *Volume (µL)* |
|---|---|
| TEMED | 20 µL |
| 10% APS (ammonium persulfate) | 400 µL |
| Total Volume | ~50,420 µL |

44. Immediately pour gel:

44.1 Use a 25 mL disposable pipette to add gel solution to the casting sandwich that was set up in Step 41. Tilt the casting sandwich on an angle and load the gel solution into the corner, slowly tilting back to even as it fills up.

44.2 Insert a custom comb that has enough wells for the control, ladders, and the oxidized and non-oxidized samples, check for air bubbles.

**NOTE: Do not insert comb all the way down into the gel.**

44.3 Allow gel to polymerize for 1-2 hrs. Periodically checking for leaks.

45. Prepare 2 L of 0.5X TBE running buffer by diluting 10X TBE with Milli-Q water.

46. Set up gel box:

46.1 Remove bottom spacer from gel and place gel sandwich in upper chamber buffer apparatus. The short gel plate should be facing away from you.

46.2 Make sure dam block is on the 2nd side of gel box if only running one gel (using another piece of the large glass, secured with clips).

46.3 Fill upper chamber with 0.5X TBE and make sure no buffer is leaking into the lower chamber. If leaking, pour out buffer (save it) and reposition gel sandwich.

46.4 Add remaining 0.5X TBE to bottom chamber and mix with a 25 mL pipette.

46.5 Carefully remove gel comb. Rinse out wells to remove urea using a large syringe with running buffer.

46.6 Check for large air bubbles under the gel and remove using large syringe with running buffer.

47. Pre run gel:

**NOTE: for all pre-runs, do not pause the run if you are not ready to continue with the protocol, the pre-run can run longer than the given amount of time**

47.1 Run gel for 15 min at 10 W.

47.2 Remove lid and rinse out wells again using syringe.

47.3 Run gel for additional 20 min at 5 W.

48. Prepare samples:

48.1 Preheat 1.5 mL block to 95°C and make sure 2X formamide loading dye is thawed.

48.2 Create a 5 µM ladder by mixing 5 µL of the **100 µM 110-nt Oligo** and 5 µL of the **100 µM 124-nt Oligo** with 190 µL of nuclease-free water.

48.3 Add 10 µL of 2X formamide loading dye to an equal volume of the oxidized sample, the non-oxidized sample, the control, and 3 ladders.

48.4 Mix tubes and place them all in 95°C heat block for 5 minutes. Then immediately place tubes in ice bucket.

49. Load samples:

49.1 Take the lid off of gel apparatus and rinse wells once again with running buffer.

49.2 Load every other lane with 20 µL of ladder/loading dye. A total of 3 ladders needed.

49.3 Load 20 µL of sample/loading dye in lanes between ladders and 20 µL of control/loading dye outside the last ladder.

50. Run gel at constant power of 7 W until xylene cyanol just visible from the back of the gel chamber and bromophenol blue reaches middle of the gel (about 2-3 hrs.).

51. Stain gel with SYBR Gold:

51.1 Thaw SYBR Gold aliquot and prepare 300 mL fresh 0.5X TBE for staining buffer.

**NOTE: SYBR Gold is light sensitive.**

51.2 Add 300 mL of 0.5X TBE and 30 µL of SYBR Gold to a PLASTIC staining dish.

51.3 Turn off the gel and remove the lid. Empty the TBE from top and bottom chamber of the gel apparatus and then remove the glass plate sandwich with your gel inside.

51.4 Carefully remove gel from sandwich using a metal spatula to pry open the two pieces of glass at the *bottom* of the gel.

**NOTE: To help remember orientation of gel, cut one corner with a razor blade.**

51.5 Place the gel into the buffer in the PLASTIC staining dish.

51.6 Cover plastic dish with aluminum foil and place on gentle shaker for 15 min.

52. Placed the newly stained gel on the blue filter box and transfer to the bio-imager to take a photograph. Select new protocol, Nucleic Acid and choose SYBR Gold. Position gel, and then “run protocol”.

53. Keep gel on blue filter box and move from bio-imager to UV light box. Turn off the lights and put on amber goggles.

54. Use a clean new razor blade to cut out the band in-between the 112nt and 124nt bands for each sample (oxidized, non-oxidized and control). Use a fresh blade for **each** sample, being careful not to contaminate blade.

**NOTE: The top band of the ladder is 2nt longer then max desired product length of 122nt.**

55. Cut each gel slice (oxidized, non-oxidized and control) into small pieces. Place the entire contents of each gel slice into its own 2 mL tube.

56. Place tubes in liquid nitrogen for about 2 minutes and then crush gel slices with DNase-free pestles.

57. Rinse pestle off into the tube with 1.6 mL of 0.3 M NaCl.

58. Place tube on shaker to nutate at room temperature overnight.

**✪★SAFE STOPPING POINT ★✪**

**Samples need to shake at room temperature overnight.**

## Day 5 – cDNA Gel Extraction, PCR, Agarose Gel, and Final Gel Product Extraction

✪ *Dry Ice is needed for freeze at step 63* ✪

### Step 11 – Extraction of cDNA from Acrylamide pieces

59. Remove samples from shaker and check to ensure gel is as dissolved as possible.

60. Transfer 400 µL of sample/gel-debris onto a Spin-X column inside a 2 mL collection tube (Sigma CLS8160) and centrifuge for 2 minutes at 13,000 rpm. Transfer that Spin-X column into a new 2 mL collection tube.

61. REPEAT step 60 until the full amount of each sample has been passed through its own Spin-X column into 4 separate collection tubes per sample.

**NOTE: the last 400 µL will be difficult to pipette cause of gel chunks. Use pipette tip as 'shovel' to help clear tube. There will now be 12 tubes**

62. To each tube add 2 µL of glycogen (20 µg/µL) and mix briefly by flicking tube.

63. To each tube add 1.2 mL of 100% EtOH, mix by inverting several times and then place on **dry ice** for 1 hr.

64. Centrifuge at 13,000 rpm for 30 min at 4°C.

65. Remove and discard supernatant and wash pellet with 750 µL 80% fresh EtOH.

66. Centrifuge at 13000 rpm for 1 min at 4°C and remove all liquid, carefully avoiding pellet.

67. Remove trace ethanol with a 10 µL pipettor and let air-dry for 3-5 minutes.

68. Resuspend the pellet in the first tube with 15 µL nuclease-free $H_20$. Then use the same 15 µL of water (now containing the resuspended pellet) to resuspend the pellet in the second, then third, and finally fourth tube.

69. Continue to **PCR Amplification** step or freeze sample.

**✪★SAFE STOPPING POINT ★✪**

**If stopping here; place sample in -20°C freezer.**

## Step 12 - PCR Amplification

70. Place 5 µL of sample into a 0.2 mL PCR tube

71. Set up the following PCR reaction per sample. Add the following components together and mix well.

| *Components* | *Volume (µL)* | *Final Conc* |
|---|---|---|
| Nuclease Free Water | 8.5 µL | |
| Template DNA | 5.0 µL | |
| 5X LongAmp Taq Reaction Buffer | 5.0 µL | 1 X |
| 10 mM dNTPs | 2.5 µL | 1 mM |
| **10 µM PCR FWD Primer** | 1.5 µL | 0.6 µM |
| **10 µM PCR REV Primer** | 1.5 µL | 0.6 µM |
| 2.5 U/µL LongAmp Taq DNA Polymerase | 1.0 µL | 0.1 U/µL |
| Total Volume | 25.0 µL | |

72. Place the samples in the thermocycler and run the PCR with following PCR cycling conditions:

| Denature | Repeat 18x | Extend | Hold |
|---|---|---|---|

| | | | | | |
|---|---|---|---|---|---|
| 94°C | 94°C | 52°C | 70°C | 70°C | 4°C |
| 30 s | 15 s | 30 s | 15 s | 5 min | ∞ |

**✪★SAFE STOPPING POINT ★✪**

**If necessary you can stop here; place sample in -20°C freezer or leave in thermocycler overnight. Ideally, continue to 2% Agarose Gel if time allows it.**

## Step 13 - Run PCR product on 2% Agarose Gel

73. Prepare a 100 mL 2% Certified Low Range Ultra Agarose gel (Bio-rad).

| *Components* | *Volume (µL)* | *Final Conc* |
|---|---|---|
| Agarose | 2 g | 2% |
| 1X TAE | 100 mL | |
| Total Volume | ~100 mL | |

74. Place the flask into the microwave and heat until boiling and agarose has melted.

75. Add 5 µL of 10 mg/mL Ethidium Bromide to the gel while it's still hot.

76. Pour gel into appropriate casting tray and insert a comb with at least 12 wells.

77. While the gel is solidifying, prepare a master mix for five 0.5 µg/lane 25 bp Invitrogen ladders. Add 2.5 µL of 1 µg/µL 25 bp Invitrogen ladder to 60 µL of nuclease free water.
**NOTE: After the 6X Loading Dye is added the loading volume of each ladder will be 15 µL.**

78. Add 5 µL of 6X Loading Dye to all four 25 µL samples. Add 12.5 µL od 6X Loading Dye to the mastermix of Invitrogen Ladder.

79. After the gel has solidified, carefully remove the comb, place in gel box and add 1X TAE until the gel is completely covered.

80. Load samples and ladders.

    80.1 Load 15 µL of the 25 bp ladder mastermix to every 3rd lane (so there are 2 open wells between each) for a total of 4 ladders.

    80.2 Load the first 15 µL of each sample into the well immediately after each ladder. Load the last 15 µL of each sample next to the first 15 µL.
    **NOTE: All samples should be aligned as shown below.**

| | | | | | | | | | | | | | | | |
|---|---|---|---|---|---|---|---|---|---|---|---|---|---|---|---|
| **C** | **L** | **PCL** | | **+** | **+** | | **PCL** | | **-** | **-** | | **PCL** | **L** | **C** | **PC** |

81. Run gel at about 70 V for 2-3 hours or until the ladder shows good separation between the 125-200bp bands.
**NOTE: May need to image the gel a few times during the run to see how far the bands have moved.**

82. When the run is complete, take a picture of the gel and then cut out the desired bands for the

oxidized and the non-oxidized samples using a clean razor blade for each cut. Each sample has two lanes so there should be two gel slices for each sample.

**NOTE: The desired product should be between 150-162 bp.**

83. Tare an empty 2.0 mL tube on the scale. Place gel slice into a 2 mL nuclease-free microcentrifuge tube. Record the weight of each gel slice.

**NOTE: If the two gel slices from the same sample have a combined weight under 350 mg they can be combined in the same tube.**

## Step 14 - Extraction of Final Product from Agarose gel via QIAGEN MinElute Gel Extraction Kit

84. Add 3 volumes of Buffer QG to 1 volume of gel (100 mg or approximately 100 $\mu$l). For example, add 300 $\mu$l of Buffer QG to each 100 mg of gel. For >2% agarose gels, add 6 volumes of Buffer QG. The maximum amount of gel slice per spin column is 400 mg; for gel slices >400 mg use more than one MinElute column.

85. Incubate at 37°C for 20 min (or until the gel slice has completely dissolved). To help dissolve gel, mix by vortexing the tube every 2–3 min during the incubation.

**IMPORTANT: Solubilize agarose completely. For >2% gels, increase incubation time.**

86. After the gel slice has dissolved completely, check that the color of the mixture is yellow (similar to Buffer QG without dissolved agarose).

**NOTE: If the color of the mixture is orange or violet, add 10 $\mu$l of 3 M sodium acetate, pH 5.0, and mix. The color of the mixture will turn to yellow. The adsorption of DNA to the membrane is efficient only at pH ≤7.5. Buffer QG contains a pH indicator which is yellow at pH ≤7.5 and orange or violet at higher pH, allowing easy determination of the optimal pH for DNA binding.**

87. Add 1 gel volume of isopropanol to the sample and mix by inverting the tube several times. For example, if the agarose gel slice is 100 mg, add 100 $\mu$l isopropanol. Do not centrifuge the sample at this stage.

88. Place a MinElute column in a provided 2 ml collection tube.. To bind DNA, apply the sample to the MinElute column, and centrifuge for 1 min. For maximum recovery, transfer all traces of sample to the column.

**NOTE: The maximum volume of the column reservoir is 800 $\mu$l. For sample volumes of more than 800$\mu$l, simply load and spin again.**

89. Discard the flow-through and place the MinElute column back in the same collection tube.

90. Add 500 $\mu$l of Buffer QG to the spin column and centrifuge for 1 min. Discard the flow-through and place the MinElute column back in the same collection tube.

91. To wash, add 750 $\mu$l of Buffer PE to the MinElute column and centrifuge for 1 min.

**NOTE: If the DNA will be used for salt-sensitive applications, such as blunt-end ligation and direct sequencing, let the column stand 2–5 min after addition of Buffer PE, before centrifuging.**

92. Discard the flow-through and centrifuge for an additional 1 min at ≥10,000 x g.

**IMPORTANT: Residual ethanol from Buffer PE will not be completely removed unless the flow-through is discarded before this additional centrifugation.**

93. Place the MinElute column into a clean 1.5 ml microcentrifuge tube.

94. To elute DNA, add 10 µl of Buffer EB (10 mM Tris·Cl, pH 8.5) or water to the center of the membrane, let the column stand for 1 min, and then centrifuge for 1 min.

**IMPORTANT: Ensure that the elution buffer is dispensed directly onto the center of the membrane for complete elution of bound DNA. The average eluate volume is 9 µl from 10 µl elution buffer volume. Elution efficiency is dependent on pH. The maximum elution efficiency is achieved between pH 7.0 and 8.5. When using water, make sure that the pH value is within this range, and store DNA at –20°C as DNA may degrade in the absence of a buffering agent. The purified DNA can also be eluted in TE buffer (10 mM Tris·Cl, 1 mM EDTA, pH 8.0), but the EDTA may inhibit subsequent enzymatic reactions.**

95. If continuing, quantify samples via Qubit 2.0 using the BR dsDNA kit.

**✪★SAFE STOPPING POINT ★✪**

**If stopping here; place sample in -20°C freezer. If time allows, quantify sample with Qubit 2.0.**

**<u>Step 15 – Quantification of Final Product via Qubit 2.0</u>**

96. Dilute 1 µL of product into 4 µL of nuclease-free water in 0.2 mL RNase-free tubes and use for quantification of DNA on Qubit 2.0.

    96.1 Set up the appropriate amount of 0.5 mL tubes for the samples plus the 2 Qubit standards.

    96.2 Prepare the Qubit working solution in a 15 mL Falcon tube by diluting the Qubit dsDNA BR Reagent 1:200 in Qubit dsDNA BR Buffer.

    96.3 Add 190 µL of Qubit working solution to each of the tubes used for standards.

    96.4 Add 10 µL of each Qubit standard to the appropriate tube.

    96.5 Add 199 µL of Qubit working solution to each of the tubes used for the samples.

    96.6 Add 1 µL of each sample to the appropriate tube.

    **NOTE: The final volume of every tube is now 200 µL.**

    96.7 Vortex every tube for 2-3 seconds, being careful to not create bubbles.

    96.8 Allow all tubes to sit at room temperature for 2 minutes

    96.9 Read Standard 1, followed by Standard 2, to calibrate the instrument.

    96.10 Read each sample to get the Assay Tube Conc (α). Then use the Qubit to calculate the Stock Conc (β). It is important to remember that Stock Conc is a 1:5 dilution of the sample. So Sample Conc (ω) must be calculated.

97) Store the remaining 8 µL of each sample in the -20°C.

**✪★FINAL STOPPING POINT ★✪**

**Place samples in -20°C until ready to submit for sequencing.**
